# New role of osteopontin in DNA repair and impact on human glioblastoma radiosensitivity

**DOI:** 10.18632/oncotarget.11483

**Published:** 2016-08-22

**Authors:** Aurélie Henry, Marie-Julie Nokin, Natacha Leroi, François Lallemand, Jérémy Lambert, Nicolas Goffart, Patrick Roncarati, Elettra Bianchi, Paul Peixoto, Arnaud Blomme, Andrei Turtoi, Olivier Peulen, Yvette Habraken, Félix Scholtes, Philippe Martinive, Philippe Delvenne, Bernard Rogister, Vincent Castronovo, Akeila Bellahcène

**Affiliations:** ^1^ Metastasis Research Laboratory, GIGA Cancer, University of Liège, Liège, Belgium; ^2^ Biology and Tumor Development Laboratory, GIGA Cancer, University of Liège, Liège, Belgium; ^3^ Department of Radiology, University Hospital Liège, Liège, Belgium; ^4^ Cyclotron Research Center, University Hospital Liège, Liège, Belgium; ^5^ GIGA Neurosciences, University of Liège, Liège, Belgium; ^6^ Department of Neurosurgery, Brain Center Rudolf Magnus Institute of Neurosciences and the T&P Bohnenn Laboratory for Neuro-Oncology University Medical Center, Utrecht, The Netherlands; ^7^ Department of Pathology, University Hospital Liège, Liège, Belgium; ^8^ Virology and Immunology Laboratory, University of Liège, Liège, Belgium; ^9^ Department of Neurosurgery, University Hospital Liège, Liège, Belgium

**Keywords:** osteopontin, glioblastoma, radioresistance, DNA damage repair, EGFRvIII

## Abstract

Glioblastoma (GBM) represents the most aggressive and common solid human brain tumor. We have recently demonstrated the importance of osteopontin (OPN) in the acquisition/maintenance of stemness characters and tumorigenicity of glioma initiating cells. Consultation of publicly available TCGA database indicated that high OPN expression correlated with poor survival in GBM patients. In this study, we explored the role of OPN in GBM radioresistance using an OPN-depletion strategy in U87-MG, U87-MG vIII and U251-MG human GBM cell lines. Clonogenic experiments showed that OPN-depleted GBM cells were sensitized to irradiation. In comet assays, these cells displayed higher amounts of unrepaired DNA fragments post-irradiation when compared to control. We next evaluated the phosphorylation of key markers of DNA double-strand break repair pathway. Activating phosphorylation of H2AX, ATM and 53BP1 was significantly decreased in OPN-deficient cells. The addition of recombinant OPN prior to irradiation rescued phospho-H2AX foci formation thus establishing a new link between DNA repair and OPN expression in GBM cells. Finally, OPN knockdown improved mice survival and induced a significant reduction of heterotopic human GBM xenograft when combined with radiotherapy. This study reveals a new function of OPN in DNA damage repair process post-irradiation thus further confirming its major role in GBM aggressive disease.

## INTRODUCTION

Glioblastoma multiforme (GBM) counts among the most aggressive solid primary brain tumors developing in humans. Currently, the first line of treatment for GBM is a surgical resection followed by radiotherapy and chemotherapy with Temozolomide (TMZ) [1]. However, and because of its high heterogeneity and its ability to diffuse through healthy brain, GBM still remains impossible to cure and the global overall survival of patients with GBM is 15 months in average [2, 3]. Despite many efforts invested in the development of anti-GBM therapies, radiotherapy remains the most applied, even if radioresistance constitutes a considerable impediment to figure out. Understanding the molecular mechanisms and identifying the targets underlying GBM extreme radioresistance remain a priority.

Osteopontin (OPN) is a phosphoglycoprotein that is a member of the Small Integrin-Binding LIgand N-linked Glycoproteins (SIBLINGs) family of extracellular matrix proteins [4]. OPN has been largely associated with the pathophysiology of cancer including cell adhesion, migration, tumor progression, development of metastasis and resistance to treatment [5–9]. Several years ago, OPN expression level was correlated with malignancy grade in GBM patients [10]. Consistently, OPN expression showed a 5-fold increase in GBM compared to lower grade astrocytoma and high OPN serum level revealed to be a poor prognosis marker for GBM patients [11].

We have previously demonstrated that OPN silencing in GBM cells decreased cell migration and inhibited tumor growth on the chicken embryo chorioallantoic membrane [12]. More recently, we have described the importance of OPN expression in the subpopulation of GBM initiating cells (GICs). Indeed, we showed that OPN silencing in GICs, isolated from U87-MG population, altered their ability to grow as spheres and to express major stem-associated transcription factors such as Sox2, Oct3/4 and Nanog. Using an orthotopic mouse model of GBM, we showed that GICs capacity to initiate tumor formation was completely abolished upon OPN silencing [13]. OPN expression has been recently associated with hypoxic regions of solid tumors which constitute a niche for highly radioresistant cancer stem cells from solid neoplasms [9]. Previous studies indicated that the silencing of OPN expression radiosensitized several cancer cell types such as lung [14] and breast [15].

In this study, we investigated the hypothesis according to which OPN expression could intervene in GBM radioresistance. We showed that OPN depletion combined to radiotherapy induced a significant shrinking of the tumors *in vivo* when compared with radiotherapy or OPN depletion alone. We demonstrated that OPN depletion in human GBM cells affected the activation of the main DNA damage response proteins. This effect was rescued by exogenous recombinant OPN indicating that secreted OPN is implicated in this process. Taken together our results indicate for the first time that OPN plays a role in the initiation of DNA repair in response to irradiation in GBM cells.

## RESULTS

### High OPN expression correlates with poor survival in GBM patients treated with radiotherapy

**Figure 1:**
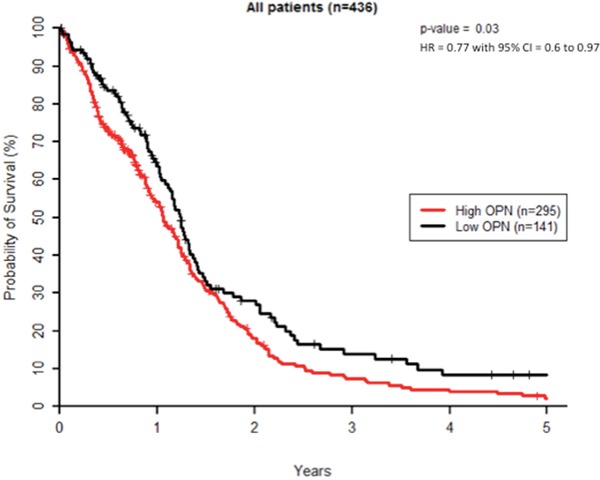
High OPN expression correlates with poor survival in GBM patients Kaplan–Meier overall survival curves for GBM patients (n=436). These results were obtained using TCGA database as described under Materials and Methods section.

We first evaluated OPN expression levels in a cohort of 438 patients from TCGA dataset for GBM. K-means clustering was used to create 2 groups of patients with the most similar OPN expression levels where 297 patients (67.8%) displayed a high OPN expression level and 141 patients (32.2%) had low OPN expression level. Homogeneity of OPN-low and OPN-high patient groups was verified for age distribution, gender, surgery, MGMT status and Karnofsky performance (KPS) scoring (Supplementary Table S1). Two patients were excluded as their survival status was unknown. We found that GBM patients with high tumoral OPN mRNA level presented a significantly lower overall survival (P<0.05) than OPN-low patients, regardless of the type of treatment (Figure 1). This observation is in good accordance with previous studies reporting that high OPN is significantly associated with poor survival in several types of cancer [16–20]. As radiation therapy is an important part of the treatment of high-grade gliomas, we addressed the question of whether the inhibition of OPN expression could affect GBM radioresponsiveness.

### OPN-depleted GBM cells are radiosensitized and accumulate more post-irradiation DNA damage than control cells

**Figure 2:**
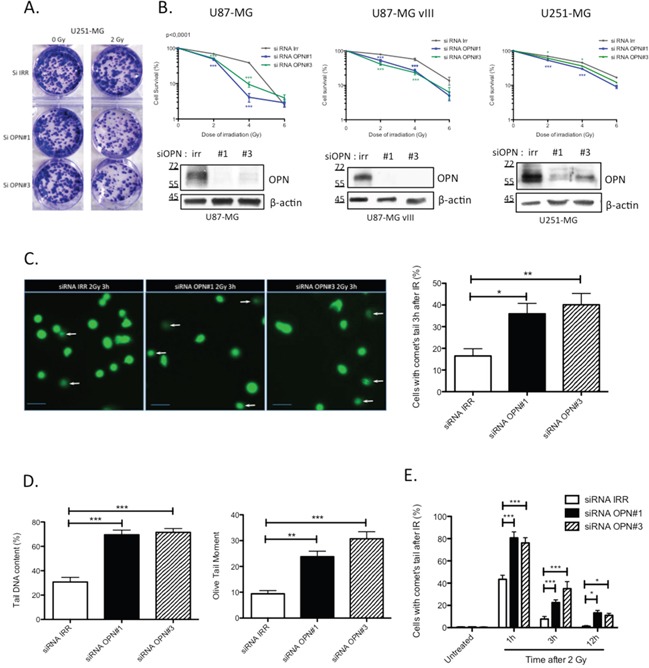
OPN inhibition affects the survival of GBM cells and delays DNA double-strand break repair **A.** Representative picture of U251-MG cells transiently transfected with siRNAs directed against OPN (siRNA OPN#1 and #3) and exposed to a single dose of 2 Gy. Cells were then submitted to clonogenic assay. Note the difference in term of colonies number when cells are depleted in OPN. **B.** Surviving fraction of U87-MG, U87-MG vIII and U251-MG was measured in OPN depleted cells compared to control after an exposition to a growing dose of irradiation ranging from 0 to 6 Gy. OPN depletion was validated by western blot. Data are presented as mean ± SEM of one representative experiment, n=3 representing 6 wells for each condition. **C.** U87-MG cells were transiently transfected with siRNAs directed against OPN (siRNA OPN#1 and #3). After 48 hours, monolayers of transfected cells were exposed to a single dose of irradiation with 2 Gy and harvested 3 hours post irradiation for comet assay. Representative cells and comet cells were imaged using epifluorescence microscopy (left panel). White arrows point to cells with comet's tail. The number of cells with comet's tail was counted for each condition (right panel). Scale bar = 50 μM. **D.** The tail DNA content and the olive tail moment were calculated, as described under Material and Methods section, in order to evaluate the amount of DNA fragments under each condition. **E.** U87-MG cells were transiently transfected with siRNAs directed against OPN (siRNA OPN#1 and #3). The number of cells with comet's tail was counted at 1h, 3h and 12h post-irradiation (2Gy). Data are presented as mean ± SEM of one representative experiment, n=3 representing 6 wells for each condition. ***, P<0.001. **, P<0.01. *, P<0.05. Immunoblot data were normalized for β-actin.

Based on the poor survival observed in OPN-high GBM patients, we explored the consequence of OPN depletion on the survival of GBM cells after irradiation. For this purpose, we used 3 GBM cell lines: U87-MG, U87-MG vIII and U251-MG cells. All 3 cell lines express and secrete OPN to different extents with U87-MG vIII cells displaying the highest level (Supplementary Figure S1A and S1B). U87-MG vIII are derived from the parental cell line U87-MG and express a constitutively active EGFR mutant. In this conformation, these cells display a higher radioresistance through the constitutive activation of the EGFR pathway [21]. We performed clonogenic assays on transiently OPN-depleted cells after exposure to growing doses of irradiation (0 to 6 Gy). We observed that OPN-depleted GBM cells formed fewer colonies compared to control as shown for U251-MG cells transfected with two siRNAs specifically directed against OPN (siRNA OPN#1 and #3) and exposed to 2 Gy (Figure 2A). Non-irradiated control cells showed a decreased number of colonies for U87-MG and U87-MG vIII upon OPN depletion while U251-MG cells did not (Supplementary Figure S1C). The calculation of cell survival fractions further demonstrated that OPN-depleted GBM cells were significantly sensitized to irradiation for all GBM cell lines analyzed when compared to control cells (Figure 2B). Consistent with their known exacerbated radioresistance [21], control and OPN-depleted U87-MG vIII showed a higher viability than parental U87-MG cells. OPN efficient depletion is shown in Figure 2B. These results indicate for the first time that OPN inhibition consistently induced GBM cells radiosensitivity. The response of cancer cells to ionizing radiation insult is strictly linked to DNA damage detection and repair capacity. To assess DNA damage response in OPN depleted GBM cells, we performed single-cell gel electrophoresis comet assay. Three hours after a single exposure to 2 Gy-irradiation, OPN-depleted cells (siRNA OPN#1 and #3) showed a higher proportion of cells with comet's tail than control cells as shown and quantified in Figure 2C. Measurement of tail DNA content and the olive tail moment confirmed that OPN-deficient cells had significantly more DNA fragments compared to control cells (P<0.0001) (Figure 2D). Next, we addressed the question whether DNA damage was persistent with time in OPN-depleted cells when compared to control cells. For this purpose, we counted the number of cells with comet's tail in non-irradiated cells and after 1h, 3h and 12h post 2 Gy-irradiation. We observed that OPN-deficient U87-MG cells presented a significantly higher number of cells with comet's tail than control cells up to 12h post irradiation (Figure 2E). The number of cells with comet's tail in non-irradiated cells did not show any significant difference between OPN-depleted and control cells. These results indicated that OPN depletion induces a delay in the resolution of DNA fragments provoked by irradiation that could be explained by impairment in the activation of the repair process.

### OPN silencing in GBM cell lines decreases H2AX phosphorylation and the activation of the main DNA repair effectors

**Figure 3:**
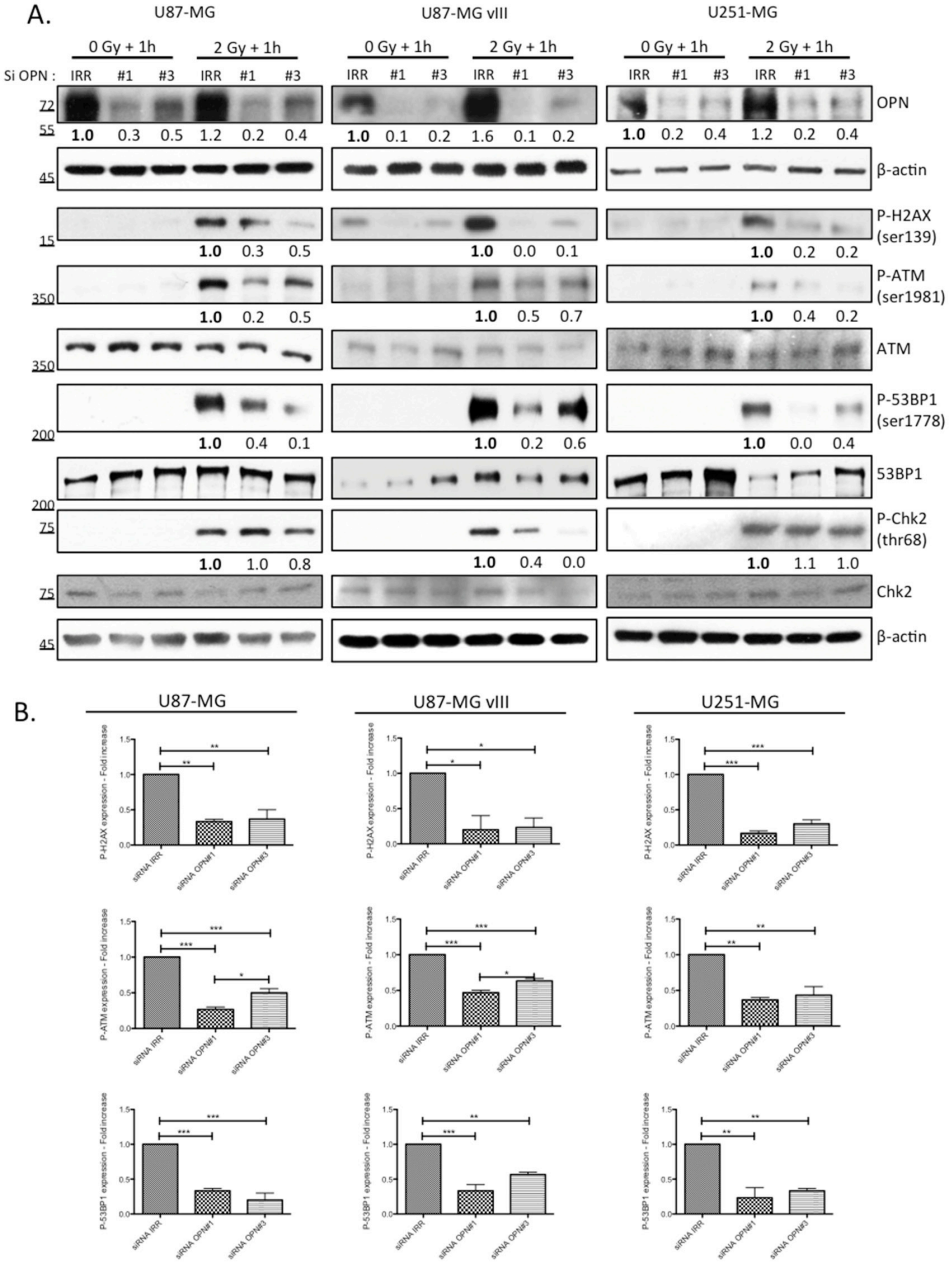
OPN inhibition affects the activation of DNA damage repair cascade in GBM cells **A.** U87-MG, U87-MG vIII and U251-MG cells were transiently transfected with siRNAs directed against OPN (siRNA OPN#1 and #3) and exposed to irradiation (2 Gy). Cells were harvested one hour post irradiation and total protein extracts were immunoblotted for phosphorylated and total DNA repair markers as indicated. Efficient OPN depletion is shown. Immunoblot data were quantified by densitometric analysis and normalized for β-actin. All immunoblots were repeated at least 3 times. **B.** Densitometric values of 3 independent experiments are represented in bar graphs as mean ± SEM for irradiated cells.

An immediate result of ionizing irradiation exposure is the phosphorylation of the histone H2A variant H2AX that forms rapidly at the sites of DNA double-strand breaks. Phosphorylated H2AX is required for the concentration and stabilization of DNA repair proteins and plays a role in both non-homologous end-joining (NHEJ) and homologous recombination (HR) repair pathways. We found that OPN-deficient cells presented less phosphorylated H2AX (P-H2AX) than OPN expressing cells (Figure 3) one hour post irradiation. Next, we compared the activation of other DNA damage response effectors in OPN-depleted GBM cells and control cells. Consistently, activating phosphorylation of ATM and 53BP1 was significantly decreased in OPN-deficient U87-MG, U87-MG vIII and U251-MG cells (Figure 3A and 3B). We observed that the phosphorylation level of Chk2, an important downstream checkpoint kinase, was induced in all cell lines. Importantly, it was decreased upon OPN depletion only in U87-MG vIII cells suggesting that the activation of Chk2 is OPN-dependent in these cells. Baseline phosphorylation of H2AX and ATM was noticed for all the cell lines under study (Figure 3A), with a consistent basal activation observed for U87-MG vIII. This was consistent with a previous report indicating that EGFR vIII overexpression in glioblastoma cells caused increased levels of ROS, DNA strand breaks accumulation, and genome instability [22].

### Recombinant human OPN efficiently re-establishes phosphorylated H2AX foci in OPN-depleted GBM cells post-irradiation

**Figure 4:**
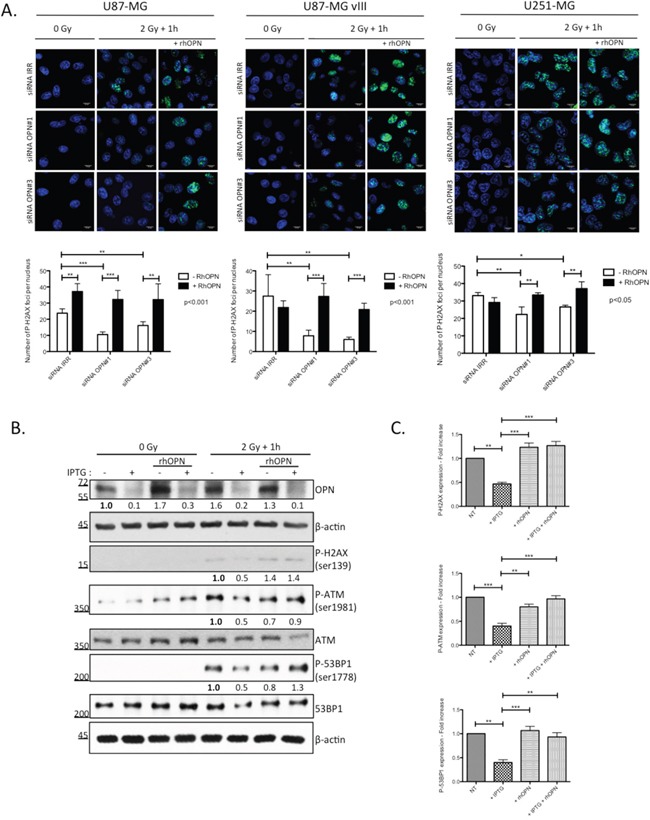
DNA damage repair markers inactivation in OPN-depleted GBM cells is rescued with soluble OPN **A.** OPN-depleted U87-MG, U87-MG vIII and U251-MG cells (siRNA OPN#1 and #3) were supplemented with exogenous human recombinant OPN (rhOPN, 250 nM) and then exposed to a single dose of 2Gy. One hour post irradiation, cells were processed for P-H2AX immunostaining (upper panel). Magnification 630x. Scale bar = 10 μM. The number of P-H2AX foci per nucleus was quantified for each condition as described under Material and Methods section (lower panel). Data are presented as mean ± SEM of one representative experiment, n=3 including 6 cover slips for each condition. **B.** IPTG-inducible shOPN U87-MG cells were maintained 4 days in IPTG-containing medium (1 mM) in order to decrease OPN expression (+ IPTG). Cells were then exposed to a single dose of irradiation of 2 Gy and harvested one hour post irradiation. Total protein extracts was immunoblotted for phosphorylated and total DNA repair markers as indicated. Immunoblot data were quantified by densitometric analysis and normalized for β-actin. All immunoblots were repeated at least 3 times. **C.** Densitometric values of 3 independent experiments are represented in bar graphs as mean ± SEM for irradiated cells. ***, P<0.001. **, P<0.01. *, P<0.05.

We have previously [12], and in this study, shown that human GBM cells secrete OPN. We next added recombinant human OPN in the culture medium of OPN-depleted GBM cells prior to irradiation and we evaluated P-H2AX foci using immunofluorescence. As expected, we found that OPN-depleted U87-MG, U87-MG vIII and U251-MG cells showed less P-H2AX foci when compared to control cells (Figure 4A). The addition of recombinant human OPN in the medium before irradiation re-established significantly the formation of P-H2AX foci. The counting of foci number in the nuclei confirmed the significant rescue of P-H2AX foci formation in presence of exogenous OPN in all 3 cell lines (Figure 4A).

We next validated our observations in IPTG-inducible shOPN U87-MG clones before their subsequent use in *in vivo* experiments. IPTG-inducible shOPN U87-MG cells were maintained in a medium containing IPTG during 2, 5 and 10 days and the efficiency of OPN silencing was assessed at the mRNA level (Supplementary Figure S2). Accordingly, IPTG addition during 4 days induced a significant decrease of OPN level as assessed by western blot (Figure 4B). Then, the cells were either non irradiated or exposed to a single dose of 2 Gy and extracted one hour after. Consistent with our previous observations using OPN siRNA silencing on P-H2AX, IPTG inducible OPN-depleted U87-MG cells demonstrated a lower level of activation of DNA repair markers (Figures 4B and 4C). We proved using this model that exogenous OPN allowed the rescue of not only the activation of H2AX but also that of ATM and 53BP1 markers (Figure 4B).

### OPN knockdown improves the survival of GBM-bearing mice and induces tumor shrinking when combined with radiotherapy

**Figure 5:**
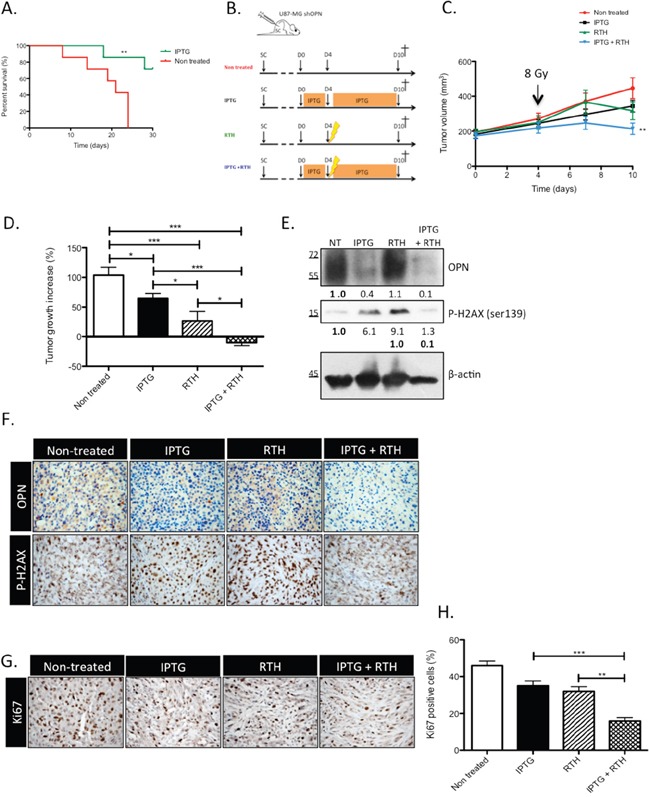
OPN knockdown improves the survival of GBM-bearing mice and induces a tumor shrinking when combined to radiotherapy by decreasing the response to irradiation **A.** Kaplan-Meier survival plot of mice bearing IPTG-inducible shOPN U87-MG orthotopic tumors. Tumors were allowed to grow during 2 weeks. Then, mice were either non treated or treated with IPTG (20 mM) by daily IP injection and supplied with IPTG-containing drinking water (10 mM) to selectively knockdown OPN expression. *n*= 7 per group. **, P<0.001. **B.** Schematic representation of the treatment plan designed for tumor-bearing mice, as described under Material and Methods section, for the heterotopic model of injection. **C.** Subcutaneous tumors generated from IPTG-inducible shOPN U87-MG cells were allowed to reach a volume of 200 mm^3^. Mice from groups IPTG and IPTG + RTH were daily treated with IPTG by IP injection (20 mM) during 4 days. At day 4, mice from groups RTH and IPTG + RTH received a single-dose of ionizing irradiation of 8 Gy. Tumor volume was measured every 3 days. *n*= 8 for IPTG and RTH + IPTG conditions; *n*= 7 for non treated and RTH conditions. **D.** For each condition, tumor growth increase was evaluated by calculating the difference between the tumor volume at day 4 post IPTG treatment and at day 10. **E.** One hour post irradiation, one mouse of each group was sacrificed and tumors excised for total extraction protein and IHC. Immunoblots for OPN and P-H2AX detection are shown. Immunoblot data were quantified by densitometric analysis and normalized for β-actin. **F.** OPN and P-H2AX IHC staining was performed on the same tumors one hour post irradiation as in E. Magnification 100x. **G.** One week post irradiation, all mice were sacrificed and tumors were excised. Ki67 IHC was performed to assess proliferation in tumors. Magnification 100x. **H.** Quantification of Ki67 positive cells for each condition. Data are presented as mean ± SEM. ***, P<0.001. **, P<0.01. *, P<0.05.

In our previous study, we demonstrated that the sole knockdown of OPN abrogated tumor growth *in vivo* [13]. In this study, the generation and validation of IPTG-inducible OPN silencing in U87-MG clones allowed for the prerequisite formation of an *in vivo* tumor in order to evaluate: (1) survival in an orthotopic set-up and (2) tumor response to radiotherapy in an heterotopic tumor model. Two weeks after orthotopic engraftment, mice were treated with IPTG to selectively knockdown OPN expression. The mice survived better (P<0.01) when OPN expression was inhibited compared to control mice (Figure 5A). We next injected sub-cutaneously IPTG-inducible U87-MG shOPN cells to explore the combined effect of OPN suppression and radiotherapy. Four weeks after subcutaneous injection of inducible U87-MG shOPN cells, mice were treated with IPTG during 4 days and received a single irradiation (8 Gy) as illustrated in Figure 5B. We and others [23, 24] have observed a slight induction of OPN expression in irradiated GBM cells therefore we chose to apply a single dose of irradiation (8 Gy) on experimental tumors. The follow-up of tumor volume showed that OPN-depleted tumors exposed to a single dose of 8 Gy (IPTG+RTH) significantly (P<0.05) slow downed their growth compared to non-treated tumors (Figure 5C). To better appreciate this effect, we measured the difference of tumor volume between day 4 (time of irradiation) and day 10 (time of sacrifice). Remarkably, OPN inhibition combined to radiotherapy induced a significant (P<0.05) tumor shrinking in mice (Figure 5D). IPTG and RTH tumors were significantly smaller when compared to non treated tumors (Figure 5D). These observations strengthened our hypothesis according to which OPN expression is important for GBM cells response to radiotherapy.

To validate *in vivo* the impact of OPN-depletion on the activation of the DDR cascade observed *in vitro*, we performed immunoblotting and immunohistochemistry on experimental tumors sampled from one representative mouse of each group sacrificed one hour post-irradiation. Efficient OPN depletion was validated in IPTG and IPTG + RTH tumors (Figure 5E). Consistent with our *in vitro* data, the level of P-H2AX in IPTG + RTH tumors was lower than in RTH tumors indicating that P-H2AX expression was impeded in absence of OPN to a level that is similar to non treated cells (Figure 5E). Intriguingly, IPTG tumors showed an increased P-H2AX while they were not subjected to ionizing radiations (Figure 5E). We excluded that this effect could be attributed to IPTG treatment by performing P-H2AX immunoblots on IPTG-treated U87-MG cells (data not shown). Elevated levels of P-H2AX may reflect an accumulation attributable to endogenous DNA damage formed for example as a consequence of oxidative stress. Indeed, a pioneer work of Denhardt and Chambers [25] suggested that high OPN-expressing cancer cells inhibit NO production thus protecting themselves against oxidative stress. Using IHC, we further observed that the staining of P-H2AX was less intense in IPTG + RTH tumors in good accordance with a role for OPN depletion in the inactivation of early DNA double-strand repair signal transducers (Figure 5F). OPN detection was decreased in IPTG and IPTG + RTH and slightly increased in RTH conditions when compared with non treated tumors (Figure 5F).

### OPN depletion *in vivo* decreases the proliferation of GBM cells when combined to radiotherapy

At day 10, all mice were sacrificed and subcutaneous tumors were sampled to perform Ki67 immunostaining to evaluate tumors proliferative rate (Figure 5G). A high rate of Ki67 positive cells was calculated in tumors collected from non-treated mice (45 to 50%) (Figure 5H). IPTG and RTH tumors showed a lower Ki67 index (20 to 40%) compared to non treated tumors. IPTG + RTH tumors showed a significantly lower proportion of Ki67 positive cells (10 to 20%, P<0.001) compared to the other conditions indicating that the inhibition of OPN expression combined to RTH decreased cell proliferation thus resulting in reduced tumor volume.

## DISCUSSION

A previous study reported a decreased clonogenicity for OPN-depleted cells after irradiation. However the role of OPN in such acquired radioresistance has not yet been fully elucidated [24]. In this study, we report for the first time the impact of OPN silencing on the radiosensitivity of GBM cells through interference with DNA double-strand repair machinery. We demonstrated that the inhibition of OPN in GBM cells impaired the activation of early signal transducers of DNA double-strand damage following ionizing radiation thus resulting in an enhanced radiosensitivity of GBM cells. Importantly, using a mouse GBM xenograft model, we have shown that the inhibition of OPN combined to radiotherapy induced significant GBM tumor shrinking when compared with OPN inhibition or radiotherapy alone.

A recent transcriptomic study, aimed at the identification of therapeutic targets for GBM, pointed to OPN as a central node between 3 main pathways: Wnt signaling, cell cycle and focal adhesion which indicated that OPN is an important player in dedifferentiation of glioma cells [26]. Glioblastoma stem cells, or initiating cells (GICs), have been shown to be involved in experimental tumorigenesis, tumor maintenance and therapeutic resistance [27]. We have recently shown that OPN expression in GICs is essential to maintain their stemness phenotype and tumorigenicity [13]. Interestingly, GICs have been reported to be highly resistant to irradiation by activation of DNA damage response [28]. In future studies using GICs it will be important to elucidate whether OPN expression could be responsible for their enhanced capacity to repair DNA damage after irradiation.

In this study, we have shown for the first time that the activity of ATM, the kinase that is rapidly and specifically activated in response to DNA double-strand breaks, is decreased in OPN-depleted GBM cells upon irradiation. As a consequence, its main downstream targets such as H2AX, 53BP1 and the check-point protein Chk2 were rendered inefficient to further trigger DNA repair. Knowing that these specific DNA damage response proteins are the main players of resistance in GBM, our data demonstrate that OPN inhibition represents a therapeutic target to counteract GBM radioresistance. Nowadays, the development of small molecule inhibitors disrupting the functions of DNA repair proteins such as ATM, 53BP1 or DNA-PK is considered as an attractive therapeutic strategy against cancer radioresistance [29, 30].

It is noteworthy that the inhibition of OPN affected significantly the survival of U87-MG vIII cells which are known to present with enhanced tumorigenicity and radioresistance [31]. In particular, U87-MG vIII cells have been described to rely on a rapid and efficient DNA repair mechanism [21, 32]. Moreover, the radioresistance of these cells is enhanced when compared to U87-MG cells and occurs mostly through the activation of DNA-PK [33–35]. In response to DNA damage, DNA-PK and ATM activate Chk2 that is central to the induction of cell cycle arrest and apoptosis. An important finding of this study is that OPN silencing decreased the phosphorylation of Chk2 in U87-MG vIII cells but not in U87-MG nor in U251-MG cells. This observation suggests that aggressive U87-MG vIII cells are OPN-dependent to control DNA-PK/ATM/Chk2 signaling.

We have previously demonstrated that OPN indirectly activates wild-type EGFR canonical pathway in U87-MG cells [13], potentially via CD44 ligation as shown for hyaluronan-CD44 interaction [36, 37]. Considering that U87-MG vIII cells present with both wild-type and truncated forms of EGFR, it is expected that they are responsive to OPN-mediated activation. Thus, U87-MG vIII cells radioresistance could be in part attributed to OPN stimulus as demonstrated by their significant decreased survival in response to OPN depletion. Exogenous OPN addition to GBM cells deprived for OPN re-established basal activation of the main DNA damage response proteins post-irradiation indicating that soluble OPN is necessary to initiate DNA repair process through binding cell surface receptors, such as integrins and CD44 receptor, on GBM cells. Pietras and collaborators [38] recently reported that OPN promotes radiation resistance in GBM cells via activation of CD44 signaling. Based on these studies and considering that both OPN and CD44 expression are part of the phenotype of aggressive GBM cells, it is tempting to speculate that OPN might interact with CD44 to mediate, at least in part, radioresistance.

In our *in silico* analysis of TCGA datasets, OPN had a significant predictive potential in estimating survival in GBM patients treated with conventional therapy. High OPN plasma levels are associated with poor outcome after radiotherapy in head and neck patients [39]. Molecular signatures associated with tumor aggressiveness and progression have been established in order to better determine the prognosis of patients diagnosed with a GBM and to adjust the choice of treatment [40]. Future studies will reinforce the cumulative evidence indicating that OPN expression in GBM is crucial in terms of patient survival, response to therapies and tumor recurrence.

## MATERIALS AND METHODS

### In silico data

Clinical data and mRNA expression level data [41] were downloaded from TCGA Portal (https://tcga-data.nci.nih.gov/tcga/). OPN mRNA expression levels were extracted from Agilent G4502A level 3 data. Expression levels were centered on the mean OPN expression level. K-means clustering was used to partition patients in two clusters –high and low expression– regarding OPN expression levels. A cohort of 438 primary GBM patients with available OPN levels was used for survival analysis with all treatment confounded. Survival differences were assessed by Kaplan-Meier curves and G-rho family of tests (“survival” package in R).

### Antibodies and reagents

Anti-OPN antibody was purchased from R&D Systems. Anti-Phospho H2AX (ser139, clone JBW301) and anti-ATM antibodies were purchased from Millipore. Anti-53BP1 was purchased from Novus Biologicals. Anti-Phospho ATM (ser1981), anti-Phospho 53BP1 (ser1778), anti-Chk2 and anti-Phospho Chk2 (thr68) antibodies were purchased from Cell Signalling. Anti-beta actin (clone AC-15) antibody was purchased from Sigma Aldrich. Anti-mouse Alexa 488 was purchased from Life Technologies. Secondary antibody rabbit anti-mouse HRP was purchased from Dako; secondary antibody donkey anti-goat HRP was purchased from Santa Cruz and secondary antibody goat anti-rabbit HRP was purchased from Invitrogen. IPTG (isopropyl β-D-1-thiogalactopyranoside) was purchased from Sigma Aldrich. Recombinant human osteopontin (full length) was a kind gift of Dr. Larry W. Fisher (National Institute of Dental and Craniofacial Research, Bethesda, MD, USA).

### Cell culture and treatment

U87-MG and U251-MG human glioma cell lines were obtained from American Type Cell Collection (ATCC), cultured in the recommended medium and maintained at 37°C in a humidified incubator with an atmosphere of 5% CO_2_. The cells were authenticated through DNA profiling of 8 different and highly polymorphic short-tandem repeat loci (DSMZ, Braunschweig, Germany). U87-MG cells expressing the auto-sufficient and truncated EGFR (U87-vIII) were a kind gift of Dr. Frank Furnari (UC San Diego School of Medicine, CA, USA). For transient silencing of OPN in GBM cell lines, calcium phosphate-mediated transfections were performed as described previously [13]. SiRNAs were synthesized by Eurogentec (Liège, Belgium). SiRNAs specific to OPN ([#1] Sense : 5′-CAC-AAG-CAG-UCC-AGA-UUA-UUU- 3′ and [#3] Sense: 5′-ACG-ACU-CUG-AUG-AUG-UAG-A-3′) were used to silence the corresponding target genes at a concentration of 40 nM for 48h. On Target Plus Control Pool Non Targeting Pool was purchased from Thermo Fisher and was used as a negative control. For selective OPN knockdown in U87-MG shOPN cells, IPTG was used at a concentration of 1 mM in culture medium for four days, by replacing medium one day out of two. *In vitro* irradiation of cells was performed using a GammaCell irradiator (137Cs source, 148 TBq, GammaCell 40 Exactor, Best Theratronics). For soluble exogenous OPN rescuing, cells were treated 30 minutes before irradiation with recombinant human OPN at a concentration of 250 nM.

### Generation of U87-MG with inducible shRNA OPN

U87-MG cells were first transformed using pLenti6-Luc plasmid that was generated by cloning the luciferase gene into the pLenti6/V5-D-Topo vector (K4955-10, Life Technologies). Following selection of the positive cells, the U87-MG-luc+ cells were further transformed using pLKO-shOPNi plasmid that was generated by cloning the shRNA sequence in the pLKO-puro-IPTG 3xLacO vector. The shRNA OPN vector (TRCN 0000342563) was purchased from Sigma. Lentiviral vectors used for the above transformations were generated by co-transfecting the Lenti-X 293T cells (Clontech) with pLenti6-Luc or pLKO-shOPNi. Lentiviral supernatants were collected 48h, 72h and 96h post transfection, filtrated and concentrated 100x by ultracentrifugation. The lentiviral vectors were then titrated with qPCR Lentivirus Titration Kit (LV900; Applied Biological Materials) and used to transduce U87-MG cells. Stably transduced U87-MG cells expressing luciferase were isolated and maintained in medium supplemented with 10 μg/mL of blasticidin (ant-bl-1; Invitrogen). Then, U87-MG-luc+ were transduced with shRNA-OPNi lentiviral vectors, selected and maintained in medium supplemented with 10 μg/mL of puromycin (ant-pr-1; Invitrogen) and 10 μg/mL of blasticidin.

### Western blot analysis

Cells from culture and tissue powder were lysed using SDS buffer (SDS 1%) containing protease and phosphatase inhibitor mixture (Roche). Equal amounts of proteins were resolved by SDS-PAGE. Membranes were probed with primary antibodies (see section Antibodies and Reagents), followed by horseradish peroxidase-conjugated secondary antibodies, and developed by chemiluminescence detection. Relative density of the bands was calculated using ImageJ software [42].

### Clonogenic assay

Forty-eight hours following transfection, 500 cells per well were seeded in a 6-well plate and allowed to adhere before being exposed to gamma-irradiation with a gamma-ray dose that ranged from 0 to 6 Gy. After treatment, cells were incubated at 37°C for 7 to 12 days, depending on the cell line. After this period, cells were washed with PBS and stained with Violet Crystal to count colonies presenting more than 50 cells. For each biological replicate, two wells were analyzed for colonies counting. The surviving fraction was then calculated based on the platting efficiency index [43].

### Comet assay

A comet assay kit (OxiSelect, 3-well slides, Cell Biolabs) was used for single cell gel electrophoresis, according to the manufacturer instructions. Briefly, 1×10^5^ cells/ml suspended in PBS were mixed with liquefied comet agarose at 1:9 (v/v) ratio. A volume of 100 μl of this mixture was then transferred to agarose-coated slides. The embedded cells were treated with a lysis buffer at 4°C for 45 minutes in the dark. Then, slides were treated with an alkaline solution containing NaOH and EDTA solution at 4°C during 30 minutes in the dark to unwind the double-stranded DNA. Afterwards, slides were electrophoresed under alkaline conditions at room temperature at 1 volt/cm for 30 minutes. Finally, cells were fixed in cold 70% ethanol. Slides were allowed to air dry and cells were stained with diluted (1/10.000) Vista Green DNA Dye at room temperature for 15 minutes. Stained cells were counted (6 wells of 3-well slides per condition) and photographed using an Evos AMG epifluorescent microscope (Thermo Scientific). Images were scored using the Image J software for comet assay parameters. These parameters were calculated by measuring the fluorescence and the length of the head and of the tail of comets. The following formulas were applied to measure *DNA content and olive tail moment (where “I” is the intensity of fluorescence):* Head DNA %: (I head/(I head + I tail))*100; Tail DNA %: 100 – head DNA %; *Tail moment length:* ((head length/2) + (tail length/2)) and *olive tail moment:* (tail moment length*tail DNA %)/100.

### Immunofluorescence

After treatment, cells were first rinsed with PBS. Afterwards, cells were fixed with pre-cold solution of methanol-acetone at -20°C for 10 minutes. Then, cells were incubated with PBS-BSA for 30 minutes. After blocking, cells were incubated with primary antibody at 4°C overnight in a humidified atmosphere. After the removal of the primary antibody, the secondary antibody was added and incubated at room temperature for 1 hour. Nuclei were labelled with Hoeschst solution and slides were mounted with Mowiol before being observed using a confocal microscope Leica SP5 (Leica). For each biological replicate (n=3), two cover slips were imaged and three fields per cover slip were photographed. Images were then analyzed by ImageJ software to count the number of P-H2AX foci in each picture. The number of foci per nucleus was obtained by dividing the total number of foci by the number of nuclei counted.

### ELISA assay

Cells were cultured during 48h and medium was collected, centrifuged at 1000g to discard any cell material and the supernatant was analyzed for human OPN level using a commercially available ELISA kit (R&D Systems) according to the manufacturer's instructions.

### Orthotopic model of GBM in mouse

All the experimental procedures and protocols involving mouse models were performed according to the Federation of European Laboratory Animal Sciences Associations (FELASA) and were reviewed and approved by the Institutional Animal Care and Ethics Committee of the University of Liège (n°13-1602, Belgium). Seventy-five thousand U87-MG shOPN cells suspended in 2 μl of PBS were injected in the right striatum of 6-weeks old female NOD-scid mice (n=8 per group). Two weeks following the tumor engraftment, silencing of OPN was induced by IPTG that was diluted in drinking water at a concentration of 10 mM during five days. In addition, mice were treated with a daily intraperitoneal (IP) injection of 200 μl 20 mM IPTG solution (or 200 μl of NaCl solution for non treated condition). Tumor growth was monitored using bioluminescence on an IVIS 200 imaging system (Xenogen-Caliper) after luciferine injection (Cat. No. E1605; Promega). Mice were daily checked for clinical signs and weighted every 2 days. All mice were sacrificed at the onset of first neurologic signs in any animal. One month post-xenografting, all surviving mice were sacrificed.

### Heterotopic model of GBM in mouse

U87-MG shOPN cells (1.2 × 10^6^) were injected subcutaneously in the right flank of 4- to 6-weeks old female NOD-scid mice in a final volume of 300 μl. Four weeks following injection, tumor size was calculated based on caliper measures and mice were divided in 4 groups of treatment plan: non-treated mice (n=7); OPN-depleted mice (IPTG) (n=8); mice treated by radiotherapy (RTH) (n=7) and OPN-depleted mice treated by radiotherapy (IPTG + RTH) (n=8). Mice belonging to groups IPTG and IPTG + RTH were treated with a daily IP injection of 200 μl 20 mM IPTG solution for 4 days (or 200 μl of NaCl solution for non treated condition). At day 4, mice from groups RTH and IPTG + RTH received a single dose of gamma radiation (8 Gy) using the irradiator X-RAD 225Cx (Precision X-Ray Inc., North Branford, CT). One hour post irradiation, representative mice of each group were sacrificed for histological and protein analysis. Thereafter, tumor growth of the remaining mice was monitored every 3 days using a caliper. Tumor volume was assessed using the formula V=4/3*π*(h/2)*(l/2)*(w/2) where h, l and w denote height, length and width, respectively. One week post-radiotherapy, all the mice were sacrificed and the tumors were prepared for histological analysis. Pieces intended to histology were fixed in PFA 4% prior the embedding in paraffin. Pieces intended to protein analysis were snap-frozen in liquid nitrogen and reduced into powder by crushing.

### Immunohistochemistry

Subcutaneous tumors were collected from euthanized mice and fixed in 4% paraformaldehyde (PFA) solution for overnight at 4°C before embedding into paraffin. Paraffin tissue blocks were then cut with the microtome in 5μm thick sections and then subjected to xylene bath and rehydratation. The sections were washed in phosphate saline buffer (PBS) with 0.25% triton X-100 and boiled in 10 mM sodium citrate buffer (pH6) using microwave for 30 min (OPN, P-H2AX) or water-bath at 95°C for 40 min (Ki67). The sections were blocked for 30 min in PBS-normal serum solution (150μL normal serum and 20μL Tween 20 (Sigma Aldrich) in 10 mL PBS) and incubated with the primary antibodies at 4°C overnight. Following antibodies were used: OPN (Abcam, dilution 1:1000), P-H2AX (ser139, Abcam, dilution 1:3000, 1h at room temperature) and Ki67 (clone MIB-1; Dako, dilution 1:100). Following this, the slides were washed once with the blocking solution and the sections were incubated with biotinylated anti-rabbit/mouse IgG (dilution 1:500) for 30 min. Subsequently, the sections were washed in PBS and incubated in avidin-biotin complex kit for 30 min. Finally, the tissue sections were stained with 3, 3′-diaminobenzidine (DAB) and counter-stained in hematoxylin. Pictures of representative fields were taken under a light microscope Leica DMRB (Leica).

### Statistical analysis

All results were reported as means with standard error of the mean (SEM) as described in figure legends. Survival curves were compared using a Log-Rank test. Comparisons between group means were performed according to one-way ANOVA followed by a pairwise comparison with Bonferroni's post-test. When needed, depending on the number of grouping factors, two-way ANOVA were performed followed by a Bonferroni's post-test. P<0.05 was considered as statistically significant. All experiments were performed in at least three independent biological replicates.

## SUPPLEMENTARY FIGURES TABLE


