# Supplementary material for: New role of osteopontin in DNA repair and impact on human glioblastoma radiosensitivity
Source: Oncotarget. 2016 Aug 22;7(39):63708–21. doi: 10.18632/oncotarget.11483 (PMC5325397; doi:10.18632/oncotarget.11483)
Supplement: Supplementary file 1 [file oncotarget-07-63708-s001.pdf]

New role of osteopontin in DNA repair and impact on human glioblastoma radiosensitivity

SUPPLEMENTARY FIGURES TABLE

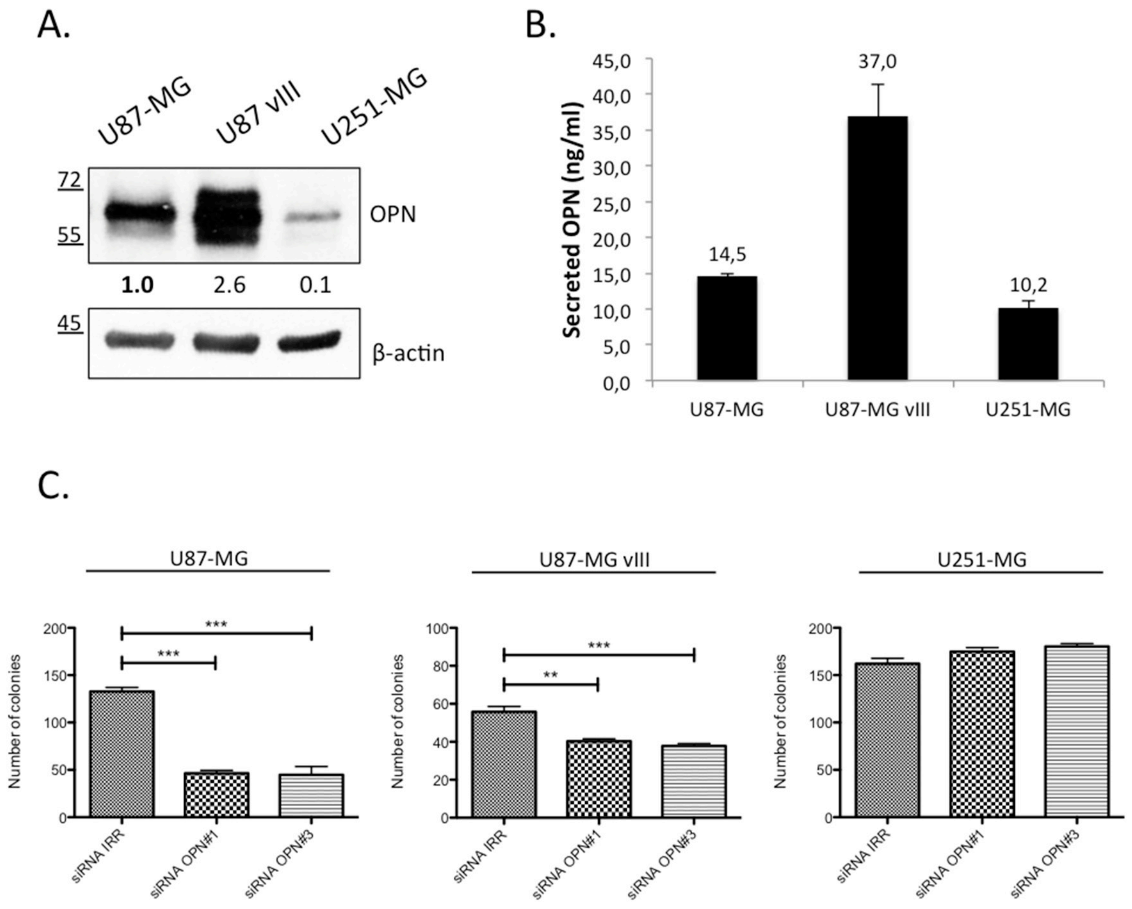

Supplementary Figure S1: OPN expression in U87-MG, U87-MG vIII and U251-MG cells and impact of OPN depletion on cell viability. OPN expression **A.** and secretion **B.** was quantified by western blot on total protein extracts and by ELISA on conditioned medium collected 48h after seeding, respectively. **C.** Graphs presenting the number of colonies at the term of the clonogenic assay upon OPN inhibition and without irradiation. Immunoblot data were quantified by densitometric analysis and normalized for β-actin. All experiments were repeated 3 times. Data are presented as mean ± SEM. ***, P<0.001. **, P<0.01.

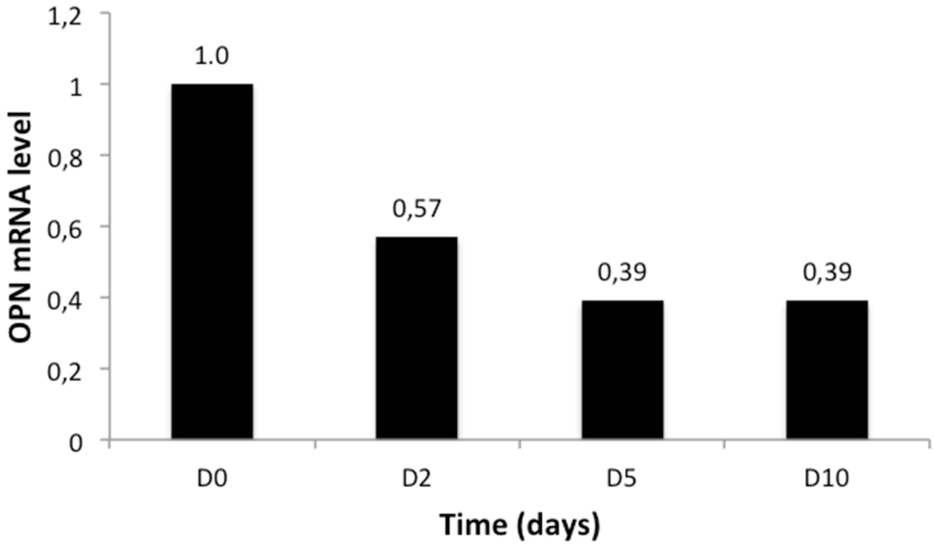

Supplementary Figure S2: OPN expression is efficiently silenced in IPTG-inducible U87-MG shOPN cells upon IPTG treatment. OPN mRNA level was quantified by qRT-PCR at day 2, 5 and 10 of treatment.

Supplementary Table S1: Characteristics of patients included in *in silico* data analysis

Factor	Category	Number of GBM patients	
		High OPN level (n=297)	Low OPN level (n=141)
Gender	Female	119	45
	Male	177	96
	Unknown	1	0
Age	< 50 years old	60	38
	50-70 years old	169	69
	> 70 years old	67	34
	Unknown	1	0
Karnofsky score	100	21	15
	90	1	1
	80	128	66
	70	3	1
	60	54	20
	40	11	3
	20	2	0
	Unknown	77	35
Surgical procedure	Biopsy	41	20
	Tumour resection	254	119
	Unknown	2	2
Additionnal treatment	RTH alone	23	17
	RTH followed by TMZ	41	20
	RTH + concomitant and adjuvant TMZ	122	64
	Other	111	40
MGMT promoter status	Methylated	100	37
	Non-methylated	101	48
	Unknown	96	56

This table summarizes the different criteria taken into account to verify the homogeneity of the two groups of GBM patients analyzed.
